# Towards Automatic Early Detection: Assessing LANGaware's Language and Speech Biomarkers in Neurocognitive and Affective Disorders

**DOI:** 10.1002/alz.088700

**Published:** 2025-01-09

**Authors:** Vassiliki Rentoumi, Evangelos Vassiliou, Dimitra Sali, Admir Demiraj, George Paliouras

**Affiliations:** ^1^ LANGaware Inc, Menlo Park, CA USA; ^2^ NCSR Demokritos, Athens Greece; ^3^ Department of Financial and Management Engineering, School of Engineering, University of the Aegean, Chios Greece; ^4^ Aiginiteio University Hospital, Athens Greece

## Abstract

**Background:**

Recent advancements in automatic language and speech analysis, coupled with machine learning (ML) methods, showcase the effectiveness of digital biomarkers in non‐invasively detecting subtle changes in cognitive status. While successfully distinguishing between Alzheimer's Disease (AD) and Normal Control (NC) individuals, classifying Mild Cognitive Impairment (MCI) proves to be a more challenging task. MCI can progress to AD or result from various factors, including affective disorders, necessitating multiple expert examinations for accurate detection. Building upon previous research, we create an experimental setup to assess LANGaware’s biomarkers pool on three objectives: a) binary separation into Dementia and NC cohorts, b) broad three‐class separation into Dementia, NC, and MCI groups, c) binary differentiation into Depression coupled with Anxiety disorder and NC cohorts.

**Method:**

Patient audio recordings and ASR‐generated transcripts were fed into LANGaware’s multimodal ML pipeline, extracting hundreds of linguistic and audio features, distilled into interpretable categories with a neural network assigning weights. These categorical values served as inputs to a final neural network layer generating probabilities for target labels (Dementia, NC). Similar methodologies were applied to our second (Dementia vs MCI vs NC) and third discrimination task (Depression/Anxiety vs NC), where the neural network allocated varying weights to input features for each of the aforementioned cases.

**Result:**

In all scenarios, data were split into a 70% training set and a 30% testing set, validated against medical expert diagnosis. For binary separation, with 2927 Dementia and 815 NC instances, the model demonstrated 89% accuracy and an 85% macro‐averaged F1 score. For three‐class separation (3752 Dementia, 1117 NC, 5993 MCI instances), the model achieved 70% accuracy and a 71% F1 score. Discriminating affective disorders (1016 Depression/Anxiety, 1630 NC instances) resulted in 71% accuracy and a 71% F1 score.

**Conclusion:**

The assessment suggests that our modelling approach aptly discerns language and speech patterns, distinguishing individuals with MCI from those with Dementia or in optimal health (NC). These outcomes contribute significantly to automatic evaluation, offering early diagnosis and timely treatment access. Our third experiment showcases the methodology's applicability in detecting affective disorders, specifically Depression and Anxiety, which may co‐occur with or precede MCI.

